# Clinical Characteristics of Patients With Re-admitted of Novel Coronavirus 2019 (nCOVID-19) in Wenzhou, China

**DOI:** 10.3389/fpubh.2021.649178

**Published:** 2021-05-12

**Authors:** Xinchun Ye, Yuping Yuan, Risheng Huang, Aiqiong Cheng, Zhijie Yu, Ziyang Huang, Rongrong Chen, Xiangao Jiang, Yuanliang Zheng, Jichan Shi

**Affiliations:** ^1^Department of Gastroenterology, Wenzhou Central Hospital, Wenzhou, China; ^2^Department of Infectious Disease, Wenzhou Central Hospital, Wenzhou, China; ^3^Department of Thoracic Surgery, Wenzhou Central Hospital, Wenzhou, China; ^4^Department of Hematology, Wenzhou Key Laboratory of Hematology, The First Affiliated Hospital of Wenzhou Medical University, Wenzhou, China

**Keywords:** COVID-19, new coronavirus pneumonia, clinical characteristic, re-admitted, commuted tomography

## Abstract

**Background:** During the COVID-19 pandemic, many patients admitted to hospital for treatment have recovered and been discharged; however, in some instances, these same patients are re-admitted due to a second fever or a positive COVID-19 PCR test result. To ascertain whether it is necessary to treat these patients in hospitals, especially in asymptomatic cases, we summarize and analyze the clinical and treatment characteristics of patients re-admitted to hospital with a second COVID-19 infection.

**Methods:** Of the 141 COVID-19 cases admitted to the Wenzhou Central Hospital between January 17, 2020, to March 5, 2020, which were followed until March 30, 2020, 12 patients were re-admitted with a second COVID-19 infection. Data was collected and analyzed from their clinical records, lab indexes, commuted tomography (CT), and treatment strategies.

**Results:** Most of the 141 patients had positive outcomes from treatment, with only 12 (8.5%) being re-admitted. In this sub-group: one (8.3%) had a fever, a high white blood cell count (WBC), and progressive CT changes; and one (8.3%) had increased transaminase. The PCR tests of these two patients returned negative results. Another 10 patients were admitted due to a positive PCR test result, seven of which were clinically asymptomatic. Compared to the CT imaging following their initial discharge, the CT imaging of all patients was significantly improved, and none required additional oxygen or mechanical ventilation during their second course of treatment.

**Conclusions:** The prognoses of the re-admitted patients were good with no serious cases. We conclude that home treatment with concentrated medical observation is a safe and feasible course of treatment if the patient returns a positive PCR test result but does not display serious clinical symptoms. During medical observation, patients with underlying conditions should remain a primary focus, but most do not need to be re-admitted to the hospital.

## Introduction

On December 8, 2019, a novel pneumonia case was discovered in Wuhan City (1). The first isolation of the coronavirus from a patient suffering from this pneumonia was isolated on January 7, 2020, and was subsequently named COVID-19 by the WHO (2). COVID-19 is now distributed globally and has become a serious pandemic, with the virus prevalent in many countries. On January 17, 2020, Wenzhou confirmed the first new case of COVID-19 pneumonia in Zhejiang Province. Wenzhou was also the first city, other than Hubei Province, to have more than 500 confirmed cases. From the first COVID-19 patient admitted on January 17, 2020, to March 5, 2020, a total of 141 infected patients were admitted to our hospital. All of these patients recovered and were discharged according to the discharge standards set by the Chinese authorities, and no deaths occurred.

As the current epidemic situation in China continues to improve, most hospital-admitted patients are recovering and being discharged; however, we have found in our own clinical work that some patients who have been discharged develop another fever or return a second positive PCR test result, leading to their re-admission to hospital. For this sub-group, especially the asymptomatic patients, there has not yet been any relevant clinical research into whether it is necessary to be re-admitted to the hospital for treatment or if a home medical observation is sufficient. Combined with the follow-up and examination results of COVID-19 patients discharged from our hospital, we summarize and analyze the clinical characteristics, treatment options, and prognosis of patients who were re-admitted to the hospital.

## Materials and Methods

### Subjects and Study Design

We performed a retrospective analysis of the 141 confirmed cases of new coronary pneumonia admitted to our hospital from January 17, 2020, to March 5, 2020, (diagnosed with a positive pharyngeal swab test for SARS-Cov-2): The median age was 46 years (34–55); 68 patients (48.2%) were female, and 73 (51.8%) were male; 48 (39.7%) had been to Wuhan in the previous 2 weeks; 13 (9.2%) had a history of smoking; 58 (41.1%) had underlying health conditions; 44 (31.2%) had low WBC, 96 (68.1%) had normal WBC, and 1 (0.7%) had a high WBC; 56 (39.7%) had low lymphocytes, with no patients displaying a high absolute lymphocyte count; 140 (99.3%) displayed infectious lesions on their commuted tomography (CT), and 25 cases (17.7%) were severe or critical. All patients displayed at least one clinical symptom and were hospitalized for an average of 22 days, during which they all were treated with antiviral drugs. The discharge criteria was that two nucleic acid tests with an interval of more than 24 h were negative and disappearance of the signs and symptoms. Patients who have been discharged develop another fever or return a second positive PCR test result with an interval of 1 week were readmitted. And patients attended a hospital follow-up 1, 2, 4 weeks after hospital discharge. This study was approved by the ethics committee of our hospital, and the informed consent of the participants was obtained.

### Observation Index

Age, gender, smoking history, history of exposure in Wuhan, number of severe and critical cases, days of hospitalization, underlying health conditions and diseases, clinical symptoms, CT characteristics, lab indexes (including chest CT, WBC, and lymphocytes), treatment plans (including oxygen inhalation, mechanical ventilation, hemodialysis, antibiotics, antiviral drugs, and hormones), and the clinical symptoms and treatment plans of the 12 re-admitted patients during their second hospitalization.

### Statistical Methods

Statistical analyses were performed using SPSS25.0 software. The quantile method was used to measure the median age of the data, and the mean ± standard deviation was used for the average length of hospitalization. All quantitative data are described as percentages (%).

## Results

### General Clinical Information

The clinical characteristics of the 141 patients during their first hospitalization are shown in Table 1. The median age of the 12 re-admitted patients was 52.5 (39.3–57.0), which is significantly higher than the median age of the initial group, which was 46.0 (32.5–54.5) (*P* < 0.001). The average length of the second hospitalization (28.2 ± 8.3) was also longer than that of the first hospitalization (21.7 ± 7.9). There were six patients (50%) with underlying diseases and seven patients (58.3%) who required oxygen therapy, but only one patient (8.3%) was a severe case, and no patients required mechanical ventilation. Seven patients (58.3%) received prophylactic antibiotics, and two patients (16.7%) received the therapy with corticosteroids for a short time. All patients received antiviral therapy, and 12 re-admitted patients did not experience a readmission within 4 weeks from discharge.

**Table 1:** Clinical characteristic of the study patients.

		**The first hospitalization**	
**Characteristics**	**All patients**	**Re-admission**	**Not re-admission**
	**(*n* = 141)**	**(*n* = 12)**	**(*n* = 129)**
Median age (IQR, year)	46.0(34.0–55.0)	52.5(39.3–57.0)	46.0(32.5–54.5)
Female sex (%)	68(48.2)	5(41.7)	63(48.8)
Smoking history (%)	13(9.2)	3(25.0)	10(7.8)
Exposure history in Wuhan <2 weeks (%)	48(39.7)	4(33.3)	44(34.1)
Severe or critical cases (%)	25(17.7)	1(8.3)	24(18.6)
Hospital stays (mean ± standard deviation)	22.2 ± 8.1	28.2 ± 8.3	21.7 ± 7.9
Coexisting disorder (%)	58(41.1)	6(50)	52(40.3)
Hypertension	37(26.2)	4(33.3)	33(25.6)
Diabetes	11(7.8)	1(8.3)	10(7.8)
Chronic liver disease	15(10.6)	2(16.7)	13(10.1)
Chronic kidney disease	2(1.4)	0	2(1.6)
Chronic obstructive pulmonary	3(2.1)	1(8.3)	2(1.6)
Malignant tumor	1(0.7)	1(8.3)	0
Symptoms (%)			
Fever	109(77.3)	8(66.7)	101(78.3)
Cough	84(59.6)	8(66.7)	76(58.9)
Sore throat	16(11.3)	3(25.0)	13(10.1)
Shortness of breath	19(13.5)	1(8.3)	18(14.0)
Diarrhea	16(11.3)	2(16.7)	14(10.9)
Medication (%)			
Oxygen inhalation	64(45.4)	7(58.3)	57(44.2)
Need ventilator	3(2.1)	0	3(2.3)
Continuous renal replacement therapy	2(1.4)	0	2(1.5)
Using glucocorticoids	19(13.5)	2(16.7)	17(13.2)
Using antibiotics	51(36.2)	7(58.3)	44(34.1)
Using antiviral			
Recombinant Human interferon α2b	141(100)	12(100)	129(100)
Arbidol tablets	112(79.4)	7(58.3)	105(81.4)
Lopinavir and Ritonavir tablets	136(96.5)	12(100)	124(96.1)

### Clinical Indexes and Chest CT

**Figure 1:**
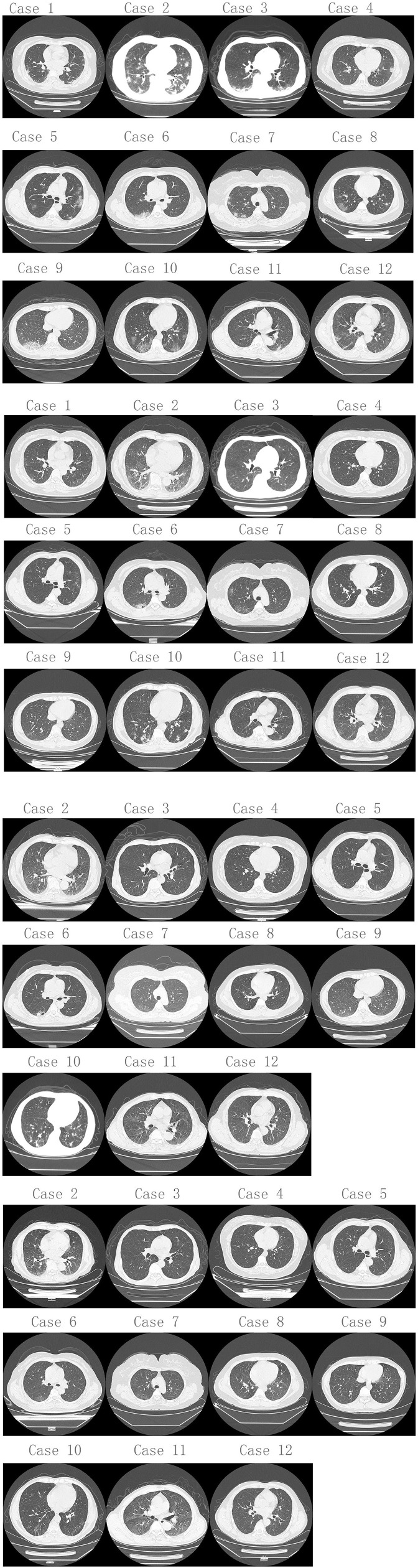
Chest CT changes from 1st to 2nd hospitalization of 12 re-admitted patients with COVID-19. **(A)** Chest CT of 1st hospitalization. Twelve patients (100%) had infectious lung lesions, mainly ground glass lesions, and both lungs were involved to varying degrees. **(B)** Chest CT of 12 re-admitted patients when 2nd hospitalized. Five patients (41.7%) still had lung infectious lesions, and only 1 patient (8.3%) had progressive lung infectious lesions compared to discharge before. The rests had much better smaller infectious lesions than discharged from hospital. **(C,D)** Chest CT changes during 2nd hospitalization of 11 re-admitted patients (except case 1).

The lab indexes and CT characteristics of the initial group of 141 patients were collected and are shown in Table 2, including first admission, discharge, and first review after their initial discharge. The detailed results of the first hospitalization of the 12 re-admitted patients are shown in Figure 1A. All of this group had infectious lung lesions, mainly ground glass lesions, and both lungs were affected to varying degrees. In three cases (25%), the absolute values of WBC and lymphocytes were lower than normal, and no patients had high WBC. The results following the first discharge of the 12 later re-admitted patients were as follows: Eight (66.7%) patients still had infectious lesions shown by CT, but they were obviously reduced compared to when they were first admitted; one (8.3%) had leukocytes levels that were lower than normal, and two (16.7%) had absolute lymphocyte values lower than normal; no re-admitted patients displayed absolute leukocyte or lymphocyte levels that were higher than normal.

**Table 2:** Radiographic and laboratory findings.

**Different stages of hospitalization**	**All patients**	**Readmission**	**Not re-admission**
	**(*n* = 141)**	**(*n* = 12)**	**(*n* = 129)**
**FIRST HOSPITALIZATION**			
Abnormalities on chest CT	140(99.3)	12(100)	128(99.2)
White-cell count			
<4.0 × 10^9^/L	44(31.2)	3(25.0)	41(31.8)
4.0–10.0 (× 10^9^/L)	96(68.1)	9(75.0)	87(67.4)
>10.0 × 10^9^/L	1(0.7)	0	1(0.8)
Lymphocyte count			
<1.1 × 10^9^/L	56(39.7)	3(25.0)	53(41.1)
1.1–3.2 (× 10^9^/L)	85(60.2)	9(75.0)	76(58.9)
>3.2 × 10^9^/L	0	0	0
**FIRST DISCHARGE**			
Abnormalities on chest CT	79(56.0)	8(66.7)	71(55.0)
White-cell count			
<4.0 × 10^9^/L	6(4.3)	1(8.3)	5(3.9)
4.0–10.0 (× 10^9^/L)	135(95.7)	10(83.3)	124(96.1)
>10.0 × 10^9^/L	0	1(8.3)	0
Lymphocyte count			
<1.1 × 10^9^/L	9(6.4)	2(16.7)	7(5.4)
1.1–3.2 (× 10^9^/L)	132(93.6)	10(83.3)	122(94.6)
>3.2 × 10^9^/L	0	0	0
**FIRST RE-EXAMINATION AFTER DISCHARGE**			
Abnormalities on chest CT	57(40.4)	5(41.7)	52(40.3)
White-cell count			
<4.0 × 10^9^/L	5(3.5)	0	5(3.9)
4.0–10.0 (× 10^9^/L)	135(95.7)	11(91.7)	124(96.1)
>10.0 × 10^9^/L	1(0.7)	1(8.3)	0
Lymphocyte count			
<1.1 × 10^9^/L	8(5.7)	2(16.7)	6(4.7)
1.1–3.2 (× 10^9^/L)	133 (94.3)	10(83.3)	123(95.3)
>3.2 × 10^9^/L	0	0	0

The results of the first examination following their initial discharge from the hospital were as follows: Five patients (41.7%) still had infectious lung lesions, and only one of these (8.3%) had progressive infectious lung lesions compared to when they were discharged, and this patient's WBC was higher than normal, suggesting that a bacterial infection may have been the cause. The others had improved with smaller infectious lung lesions than when discharged and WBC values that had returned to normal. The details of the patients during the second hospitalization are shown in Figure 1B, and the CT imaging of the 11 re-admitted patients through both hospitalizations are shown in Figures 1C,D (except case 1).

## Discussion

As of 17 March 2021, more than 120 million cases of COVID-19 infection and more than 2 million deaths have been reported globally (3). The pressure this places on the medical systems of countries with especially high infection rates is in desperate need of alleviation, which requires a coordinated international response. Other than the requirement of increased medical investment, formulating discharge standards in accordance with the national standards of each country can reduce the number of hospitalized patients and alleviate the current pressure on medical resources.

With advances in the treatments available leading to an increasing number of patients recovering from COVID-19 infections, the management of patients following discharge will become a future focus in handling the pandemic, including the follow-up time, follow-up testing protocols, and at what stage patients can return to normal life. China's current diagnosis and treatment guidelines recommend re-examining patients at 2 and 4 weeks following their discharge from the hospital. The follow-up examination protocol generally includes a routine blood test, throat swab PCR test, and a chest CT scan. The follow-up times of patients in our hospital after they are discharged are at weeks 1, 2, and 4. After reviewing the 12 re-admitted patients discussed in this study, no clinical complications occurred during the home or concentrated medical observation period, and six (50%) patients had more than one underlying health condition. Patients with underlying health conditions have a higher likelihood of being re-admitted, and so more attention should be paid to them.

Following their initial discharge, only one patient had a fever; however, the patient's chest CT lesions have progressed from the time of discharge, and the WBC, neutrophils, and c-reactive protein were significantly increased, which may have been caused by a secondary bacterial infection as the patient returned a negative PCR test result following re-admission. Another patient had nausea and vomiting symptoms, and their transaminase levels were significantly increased when they returned to the hospital; however, multiple PCR tests returned negative results following re-admission, which is unsurprising following the longer course of antiviral drugs the patient received during the first hospitalization due to a history of chronic hepatitis B. These two patients were suffering from other diseases, which suggests that although patients may recover from COVID-19 infections, their immunity may remain impaired; therefore, patients suffering from underlying diseases should pay more attention to rest, which can be monitored via close attention during medical observation (4).

The remaining 10 patients returned positive PCR tests during their re-examination, of which only three had a cough. None of these patients received additional oxygen as they all displayed blood oxygen saturation of higher than 95%, their WBC and lymphocytes had returned to normal, and their chest CT inflammation was significantly improved compared to when they were discharged from the hospital. On this basis, we recommend that if a patient is not suffering from shortness of breath, a fever, a cough, or increased sputum during the observation period following their initial discharge, there is no need to go to the hospital for a weekly review. This is especially important in regions or countries with limited medical resources.

Because pneumonia caused by COVID-19 is a novel type of viral pneumonia, our understanding of the progression of the disease needs to be improved. There remains no current evidence to say that patients who have recovered cannot infect others, so it is recommended that medical observation is still required for 14 days following discharge. According to a recent report, while there may be no live virus in sputum 8 days after the appearance of symptoms, it may remain present in the stool and blood (5). In our study, the average length of the first hospitalization was 22 days, with no patients staying <8 days. Out of 141 patients, only 12 (8.5%) were re-admitted, and of these, only two (1.4%) had worsened symptoms, though not life-threatening, which were probably caused by secondary bacterial infections. The remaining 10 patients returned positive PCR test results but were not infectious. This finding is consistent with another study that reported patients may still carry the virus and return positive PCR tests 5–13 days following discharge, though they do not infect family members contained with them during the home quarantine period (6). Patients who remain symptom-free for 14 days and receive a negative PCR test result should return to normal life, and it is safe to choose either home or centralized medical observation. According to the follow-up of this study, there have been no reports of the patients involved testing positive again for COVID-19 or infecting others, but it is recommended that patients should continue to wear protective masks and avoid going to places with crowds and/or poor ventilation.

Infected patients who are asymptomatic or displaying only mild symptoms present a high risk to the management of the pandemic and may be the most infectious group (7). To improve the management of asymptomatic infections, on April 6, 2020, the Chinese State Council issued the “Protocol for the management of novel coronavirus asymptomatic infected persons.” This states that asymptomatic infected patients must quarantine for 14 days and turned to be confirm cases once the patients presented the symptoms related to COVID-19 during the quarantine period. After the 14-day quarantine, PCR tests must be taken at 24-h intervals, and once two consecutive negative results are received, then the quarantine can end; otherwise, the quarantine must be extended until this condition is met.

One study has reported that the use of glucocorticoids is not recommended at present, and severe patients need to be especially cautious when using low to medium dose short-term treatments of glucocorticoid (8). Another study has shown that there is currently no evidence to suggest that hormone therapy could benefit patients with COVID-19 pneumonia and may actually increase the associated risks (9). However, Horby et al. showed that the use of dexamethasone in hospitalized patients is associated with lower mortality rate and better prognosis (10). And National Institutes of Health (NIH) has also emphasized the role of corticosteroids in lowering mortality rate of COVID-19 patients (11). Of the 141 patients in our study, only 10 (7.1%) were prescribed hormones for a short period, with no serious adverse reactions. Among the 12 patients who were re-admitted to the hospital, only two (one with a positive PCR test and one with a negative PCR test) received the treatment with corticosteroids for a short period of time.

In the early stage of the pandemic, some researchers suggested that since the proportion of patients re-admitted was so high the discharge standards were not sufficiently rigorous (12). The current discharge standard in China requires two consecutive negative PCR tests with an interval of more than 24 h, the significant improvement of the respiratory symptoms, a return to normal body temperature for more than 3 days, and CT imaging showing that associated lesions have improved significantly (13); however, according to this discharge standard, we find that the average length of hospitalization is too long, as many patients have no obvious symptoms in the late stage of hospitalization and their CT imaging has also improved—only a positive PCR test restricts them from being discharged. Indeed, all the re-admitted patients to our hospital had good prognoses, and none of them had related complications. In an effort to alleviate the serious pressures on medical systems in many countries due to the pandemic, we must consider lowering discharge standards. We suggest that when a patient shows significant improvements in their clinical symptoms, they be considered for early discharge, allowing them to return home where continued medical observations and monitoring of general vital signs can be made, if possible. Furthermore, this approach may also be relevant to countries who controlled the pandemic well so that they can divert their medical resources toward controlling the asymptomatically infected, which may help to control the source of future infections. This recommendation is only based on the analysis of the limited case data in our hospital, and a specific plan still needs to be comprehensively considered in accordance with the national conditions of each country and the respective severity of their epidemics.

The current results should be considered in light of a few limitations. Firstly, The sample size of this study was small and having a bigger sample size would improve the accuracy of the results. Secondly, patients were followed up at the short time interval after their first COVID-19 admissions. It has been reported that some cases were described in a time frame of up to 90 days from acute illness, and may represent persistent/fluctuant viral shedding with persistent or recurring clinical illness, rather than true reinfection (14). Finally, regarding to the possibility of reinfection or relapse of the disease in my cases, owing to no viral genome test was conducted in our study, so it is impossible to distinguish strictly. The viral RNA sequencing from both episodes showing different strains may be considered reinfection, and no new exposure and area of low community spread may be considered relapse.

In conclusion, countries with serious epidemics and tight medical resources may want to consider formulating alternative discharge standards, in accordance with national requirements, for patients whose clinical symptoms, lab indexes, and CT imaging results have improved significantly but still return positive PCR test results. These patients could be discharged from the hospital but remain under home or concentrated medical observation for 14 days. If following this period the patient receives negative PCR test results, they may return to normal life, but it is recommended that they should continue to wear protective masks and avoid going to places with crowds and/or poor ventilation; however, if the patient continues to receive positive PCR test results, while they do not need to be re-admitted to hospital, they must continue to receive 14 days of home or concentrated medical observation. This is a safe, feasible, and effective method, which could reduce the strain on medical resources.

## Data Availability Statement

The original contributions presented in the study are included in the article/supplementary material, further inquiries can be directed to the corresponding author/s.

## Ethics Statement

The studies involving human participants were reviewed and approved by the ethics committee of Wenzhou Central Hospital. The patients/participants provided their written informed consent to participate in this study. Written informed consent was not obtained from the individual(s) for the publication of any potentially identifiable images or data included in this article.

## Author Contributions

XY and YY conceived and designed the study and wrote the original draft. RH and AC collected data of epidemics, clinical features, laboratory indexes, Chest CTs, and Electrocardiography. YZ, ZH, and RC did data analysis, XJ support funding, YZ lead the project administration, and JS super revised manuscript and supervised the work. All authors contributed to the article and approved the submitted version.

## Conflict of Interest

The authors declare that the research was conducted in the absence of any commercial or financial relationships that could be construed as a potential conflict of interest.
